# Trastuzumab deruxtecan in patients with locally advanced or metastatic HER2-positive gastric cancer: a multicenter, open-label, expanded-access study

**DOI:** 10.1007/s10147-023-02422-x

**Published:** 2023-11-14

**Authors:** Kohei Shitara, Kensei Yamaguchi, Kei Muro, Hisateru Yasui, Daisuke Sakai, Takashi Oshima, Masahiro Fujimura, Yuta Sato, Shunsuke Yamazaki, Tatsuya Wakabayashi, Masahiro Sugihara, Takahiro Kamio, Hirokazu Shoji

**Affiliations:** 1 National Cancer Center Hospital East, Kashiwa, Chiba 277-8577 Japan; 2 The Cancer Institute Hospital of Japanese Foundation for Cancer Research, Koto-ku, Tokyo, 135-8550 Japan; 3 Aichi Cancer Center Hospital, Nagoya, Aichi 464-8681 Japan; 4 Kobe City Medical Center General Hospital, Kobe, Hyogo 650-0047 Japan; 5 Osaka University Hospital, Suita, Osaka 565-0871 Japan; 6 Kanagawa Cancer Center, Yokohama, Kanagawa 241-8515 Japan; 7 Daiichi Sankyo Co., Ltd, Chuo-ku, Tokyo, 103-8426 Japan; 8 Daiichi Sankyo, Inc, Basking Ridge, New Jersey 07920 USA; 9 National Cancer Center Hospital, Chuo-ku, Tokyo, 104-0045 Japan

**Keywords:** Expanded-access program, Priority review designation system, Gastric cancer, Trastuzumab deruxtecan

## Abstract

**Background:**

Trastuzumab deruxtecan (T-DXd) is an antibody–drug conjugate that consists of an anti-human epidermal growth factor receptor 2 (HER2) antibody bound by a cleavable tetrapeptide-based linker to a cytotoxic topoisomerase I inhibitor. Prior to marketing approval in Japan in September 2020, this expanded-access study was conducted to provide T-DXd to previously treated patients with locally advanced or metastatic HER2-positive gastric or gastroesophageal junction adenocarcinomas.

**Methods:**

This multicenter, open-label, expanded-access study was conducted between March 25 and September 25, 2020 at 17 Japanese sites. Previously treated patients with locally advanced or metastatic HER2-positive gastric or gastroesophageal junction adenocarcinomas received T-DXd 6.4 mg/kg via intravenous infusions at 3-week intervals. Serious adverse events (SAEs), all potential cases of interstitial lung disease (ILD)/pneumonitis, all liver-related events potentially meeting Hy’s Law criteria, and all cases of overdose were reported on the case report forms.

**Results:**

A total of 64 patients were treated with T-DXd. Among the 17 (26.6%) patients with reported SAEs, 10 (15.6%) had SAEs related to T-DXd treatment. Febrile neutropenia was the most common SAE (*n* = 6). SAEs led to death in six patients; drug-related SAEs (sepsis and febrile neutropenia) led to death in one patient. Drug-related ILD, as determined by the external Adjudication Committee, occurred in three patients (Grade 1, Grade 2, and Grade 3: all *n* = 1).

**Conclusion:**

This expanded-access study provided T-DXd to a broader population of Japanese patients prior to marketing approval in Japan, bridging the gap between clinical trials and drug approval. No new safety concerns were identified.

## Introduction

Approximately 15% of patients with gastric cancer have human epidermal growth factor receptor 2 (HER2)-positive status [1–6]. Overexpression of HER2 starts a signaling cascade that results in tumor progression [7, 8], and thus, HER2 is an important target for treating gastric cancer [7, 9]. Until recently, trastuzumab was the only anti-HER2 therapy widely approved for patients with HER2-positive gastric cancer, and it is recommended as first-line therapy in combination with standard chemotherapy treatment for this indication [10–12]. However, resistance to trastuzumab therapy has necessitated the development of other anti-HER2 treatment options for patients who do not respond or who stop responding to trastuzumab therapy.

Trastuzumab deruxtecan (T-DXd) is an antibody–drug conjugate that consists of a humanized, monoclonal, anti-HER2 antibody bound by a cleavable tetrapeptide-based linker to a cytotoxic topoisomerase I inhibitor [13]. The cytotoxic payload is membrane permeable and has a bystander effect on neighboring tumor cells [14]. T-DXd has been approved for the treatment of metastatic HER2-positive and -low breast cancer, metastatic HER2-positive gastric cancer, and metastatic HER2-mutant non-small cell lung cancer in many countries, including (but not limited to) the United States, Japan, and Europe [15–17].

Approval of T-DXd for the treatment of HER2-positive advanced gastric cancer in Japan was based on the results from the DESTINY-Gastric01 (DG-01) phase II clinical trial [18]. The DG-01 trial investigated the efficacy and safety of 6.4 mg/kg T-DXd (*n* = 125) compared with chemotherapy (*n* = 62; 55 patients received irinotecan and 7 patients received paclitaxel) in previously treated patients with HER2-positive advanced gastric or gastroesophageal junction (GEJ) adenocarcinomas. The objective response rate was significantly higher in the T-DXd cohort than in the chemotherapy cohort (51% vs 14%; *p* < 0.001); furthermore, overall survival was significantly longer than with chemotherapy (median 12.5 months vs 8.4 months; hazard ratio for death, 0.59; 95% confidence interval [CI]: 0.39–0.88; *p* = 0.01) [18]. The safety profile of T-DXd was generally manageable and consistent with an earlier phase I study: commonly reported drug-related adverse events (AEs) in both studies were hematologic and gastrointestinal in nature [18, 19]. In the DG-01 trial, 12 patients treated with T-DXd had drug-related interstitial lung disease (ILD) or pneumonitis (of which nine were Grade 1 or 2 and three were Grade 3 or 4), and there was one drug-related death (due to pneumonia) [18].

Expanded-access programs (EAPs) provide investigational drugs to patients with serious conditions and can be conducted in Japan in cases where the benefits are likely to outweigh the risks [20]. Per the Japanese EAP system, applicable investigational drugs must be intended for use to treat serious diseases, with a high degree of societal need for treatment, and for which no effective conventional treatment is available [21]. In Japan, EAPs may be initiated during or after late-phase development and can be conducted under Japanese Good Clinical Practice regulations until marketing approval or until the investigational drug is withdrawn from development. The Japanese Sakigake (meaning pioneer or forerunner) designation promotes research and development and enables early practical application of innovative medical products by conducting priority consultations, assessments, and reviews. As T-DXd was expected to have a clinical benefit for patients with previously treated HER2-positive advanced gastric cancer, it was granted Sakigake designation by the Japan Ministry of Health, Labour and Welfare (MHLW) on March 27, 2018, following the Breakthrough Therapy and Fast Track designation for HER2-positive metastatic breast cancer granted by the United States Food and Drug Administration; thus, T-DXd was considered to be a drug with high societal demand [22]. In addition, the benefits of T-DXd treatment of these patients were expected to outweigh the risks. Therefore, in accordance with the Japanese EAP system, a multicenter, open-label, expanded-access study was conducted for patients with locally advanced or metastatic HER2-positive gastric or GEJ adenocarcinoma between March 25 and September 25, 2020, prior to marketing approval in Japan.

## Patients and methods

### Study design

This multicenter, open-label, expanded-access study (DS8201-C-J4001; JapicCTI-205234; jRCT2080225138) was conducted to provide T-DXd to patients with locally advanced or metastatic HER2-positive gastric or GEJ adenocarcinoma. This expanded-access study was sponsored and conducted by Daiichi Sankyo Co., Ltd. between March 25, 2020 and September 25, 2020 at 17 Japanese sites. These study sites were also part of the DG-01 trial [18]. This expanded-access study was to be terminated when T-DXd received an approval notice for the treatment of patients with locally advanced or metastatic HER2-positive gastric cancer or GEJ adenocarcinoma; approval was received on September 25, 2020 in Japan, at which time study procedures were halted.

The expanded-access study was conducted in accordance with the ethical principles that have their origin in the Declaration of Helsinki and Good Clinical Practice guidelines, the Pharmaceuticals and Medical Devices Act (November 25, 2014), and the Japan Ministry of Health and Welfare Ordinance no. 28 on Standards for Conduct of Clinical Trials (March 27, 1997). Patients were informed about the purpose of the expanded-access study, and written informed consent was obtained.

### Patients

Eligible patients were aged ≥ 20 years; had pathologically documented locally advanced or metastatic HER2-positive gastric or GEJ adenocarcinoma (immunohistochemistry score of 3 + , or immunohistochemistry score of 2 + and positive results on an in situ hybridization assay); Eastern Cooperative Oncology Group performance status of 0 or 1; a left ventricular ejection fraction of ≥ 50% by either echocardiogram or multigated acquisition scan within 28 days of registration. The inclusion criteria for this expanded-access study were modified from the DG-01 trial to allow enrollment of patients who were assigned to the control arm (irinotecan and paclitaxel) in the DG-01 trial [18].

Patients were excluded if they had previous or current myocardial infarction or congestive heart failure; previous non-infectious ILD/pneumonitis that required steroids; current ILD/pneumonitis; suspected ILD/pneumonitis that could not be ruled out by imaging at screening; spinal cord compression or clinically active central nervous system metastases requiring steroids or anticonvulsants; or primary malignancies other than gastric and GEJ adenocarcinomas within the past 3 years.

### Treatments

Patients received T-DXd 6.4 mg/kg via intravenous infusion at 3-week intervals; this dosage was determined based on the DG-01 trial setting [18]. Two dose reductions to 5.4 mg/kg and 4.4 mg/kg were allowed per the T-DXd administration guidelines; if further dose reductions were required, the affected patients were withdrawn from the study and the treatment discontinued. The treatment continued until disease progression, unacceptable toxicities, or at study discontinuation following the marketing approval of T-DXd for metastatic HER2-positive gastric cancer in Japan.

### Study measures

Patient baseline characteristics were collected, including age, sex, primary tumor site, HER2 status (determined locally), and previous treatment histories. All serious AEs (SAEs) were captured in the case report forms (CRFs). In addition, all potential ILD/pneumonitis regardless of severity and grade, all liver-related events potentially meeting Hy’s Law criteria regardless of severity and grade, and all cases of overdose were collected. Any cases of suspected ILD were independently evaluated by an external Adjudication Committee. The criteria for potential Hy’s Law liver-related events were defined as elevated post-baseline alanine transaminase or aspartate transaminase at least three times the upper limit of normal (ULN) and an elevated total bilirubin at least 2 × ULN, either simultaneously or occurring at different time points during the study. Efficacy measures, overall survival, and post-treatment information were not collected as part of this expanded-access study.

### Statistical analyses

The enrolled analysis set included all patients who provided written informed consent and were enrolled in the expanded-access study. The safety analysis set included all patients who received at least one dose of T-DXd. As an expanded-access study, the sample size was not predetermined. However, approximately 100 enrollments were anticipated based on the geographic location of the 17 sites, experience of patient enrollment through the DG-01 trial, and an expected study duration of 7 months. Summary statistics (mean, median, standard deviation, range, and quartiles) were calculated using SAS version 9.4 or later (SAS Institute, Inc.; Cary, NC, USA).

## Results

### Patients

A total of 72 patients provided informed consent and were screened for eligibility at 17 sites in Japan, and 64 patients were enrolled in the expanded-access study. These patients received T-DXd treatment and were evaluated for safety between March 25, 2020 and September 25, 2020. All patients (*N* = 64) completed the study.

**Fig. 1:**
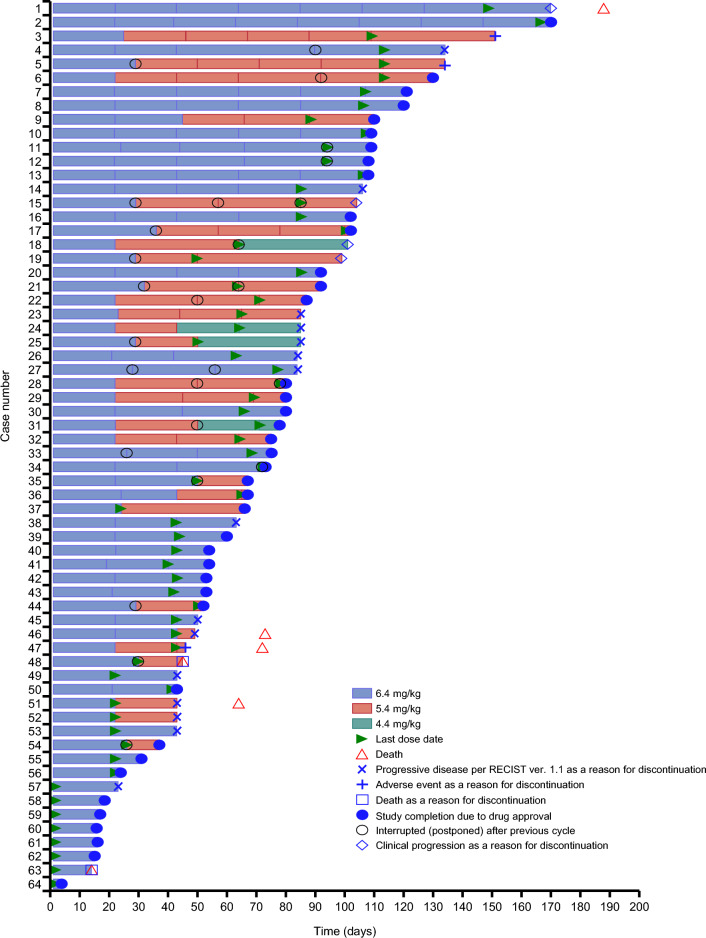
Swimmer plot indicating clinical outcomes for patients with locally advanced or metastatic HER2-positive gastric or GEJ adenocarcinoma treated with trastuzumab deruxtecan. Dosage of trastuzumab deruxtecan over time is indicated by colored bars; symbols indicate clinical outcomes. The case numbers do not reflect the order in which the cases were reported. *GEJ* gastroesophageal junction; *HER2* human epidermal growth factor receptor 2; *RECIST* response evaluation criteria in solid tumors

The patient baseline characteristics are shown in Table 1. The median age of patients was 65.5 years (range, 37–84), and the majority were male (50/64; 78.1%). Fifty-two patients had gastric adenocarcinoma (81.3%) and 12 had GEJ adenocarcinoma (18.8%). All patients had received 2 or more previous treatment regimens and the majority (37/64; 57.8%) had received four or more previous regimens, with a median (range) of previous regimens of 4 (2–11). The most common previous treatment regimens were those containing trastuzumab (*n* = 64; 100.0%), taxanes (*n* = 59; 92.2%), ramucirumab (*n* = 56; 87.5%), and immune checkpoint inhibitors (*n* = 34; 53.1%). The median duration of T-DXd treatment was 2.7 months (range, 0.7–6.2) (Table 2). All patients ultimately discontinued treatment (Table 3). The majority of patients discontinued treatment because the expanded-access study was completed as a result of drug approval (40/64; 62.5%). The dosage of T-DXd for each cycle is tabulated in Table 4, and the treatment of each patient with T-DXd is shown in Fig. 1. Twenty-seven patients (42.2%) underwent a dose reduction from 6.4 to 5.4 mg/kg, and 4 patients (6.3%) had a further dose reduction to 4.4 mg/kg. The main reason for dose reductions was AEs (27/64; 42.2%).

**Table 1:** Patient characteristics and treatment

	Total (*N* = 64)
Sex	
Female	14 (21.9)
Male	50 (78.1)
Age, years	
Mean (standard deviation)	64.4 (9.7)
Median (min, max)	65.5 (37, 84)
< 65	31 (48.4)
≥ 65	33 (51.6)
Previous cancer treatment regimens^a^	
Median (min, max)	4 (2, 11)
2	17 (26.6)
3	10 (15.6)
4	15 (23.4)
5	15 (23.4)
≥ 6	7 (10.9)
Previous treatment	
Therapy containing trastuzumab	64 (100.0)
Therapy containing taxanes	59 (92.2)
Therapy containing ramucirumab	56 (87.5)
Immune checkpoint inhibitor	34 (53.1)
Nivolumab	32 (50.0)
Irinotecan or other topoisomerase I inhibitor	21 (32.8)
Trifluridine and tipiracil hydrochloride	20 (31.3)
HER2 baseline status (before trastuzumab deruxtecan treatment)	
IHC 3 +	47 (73.4)
IHC 2 + with ISH-positive	17 (26.6)
Primary tumor site	
Stomach	52 (81.3)
GEJ	12 (18.8)

Data are shown as *n* (%) unless otherwise stated

*GEJ* gastroesophageal junction; *HER2* human epidermal growth factor receptor 2; *IHC* immunohistochemistry; *ISH *in situ hybridization

^a^Excluding radiotherapy and surgery; the expanded-access study protocol mandated that at least two prior regimens were necessary for inclusion

**Table 2:** Trastuzumab deruxtecan treatment regimen

	Total (*N* = 64)
Duration of treatment, months	
Median (min, max)	2.7 (0.7, 6.2)
0–3	43 (67.2)
> 3–6	20 (31.3)
> 6–9	1 (1.6)
Number of doses	
Mean (standard deviation)	3.6 (1.73)
Median (min, max)	3.0 (1, 9)
Quartile 1, Quartile 3	2.0, 5.0

Data are shown as *n* (%) unless otherwise stated

**Table 3:** Reasons for discontinuation of treatment

	Total (*N* = 64)
Study completion due to drug approval	40 (62.5)
Progressive disease per RECIST version 1.1	15 (23.4)
Clinical progression	4 (6.3)
AEs	3 (4.7)
Death	2 (3.1)

Data are shown as *n* (%)

*AEs* adverse events; *RECIST* response evaluation criteria in solid tumors

**Table 4:** Dose of trastuzumab deruxtecan in each cycle

	Total (*N* = 64)
Cycle 1	
6.4 mg/kg	64 (100.0)
5.4 mg/kg	0 (0.0)
4.4 mg/kg	0 (0.0)
Cycle 2	
6.4 mg/kg	33 (51.6)
5.4 mg/kg	23 (35.9)
4.4 mg/kg	0 (0.0)
Cycle 3	
6.4 mg/kg	25 (39.1)
5.4 mg/kg	18 (28.1)
4.4 mg/kg	4 (6.3)
Cycle 4	
6.4 mg/kg	17 (26.6)
5.4 mg/kg	12 (18.8)
4.4 mg/kg	2 (3.1)
Cycle 5	
6.4 mg/kg	12 (18.8)
5.4 mg/kg	5 (7.8)
4.4 mg/kg	0 (0.0)
Cycle 6	
6.4 mg/kg	6 (9.4)
5.4 mg/kg	3 (4.7)
4.4 mg/kg	0 (0.0)
Cycle 7	
6.4 mg/kg	2 (3.1)
5.4 mg/kg	0 (0.0)
4.4 mg/kg	0 (0.0)
Cycle 8	
6.4 mg/kg	2 (3.1)
5.4 mg/kg	0 (0.0)
4.4 mg/kg	0 (0.0)
Cycle 9	
6.4 mg/kg	1 (1.6)
5.4 mg/kg	0 (0.0)
4.4 mg/kg	0 (0.0)

Data are shown as *n* (%)

### Safety analysis

All 64 patients who received T-DXd were included in the safety analysis set. SAEs occurred in 17 (26.6%) patients, of whom 10 (15.6%) had SAEs related to the study drug (Table 5). SAEs led to death in six patients (9.4%); five (7.8%) of these were due to disease progression, and one (1.6%) was due to drug-related SAEs (sepsis and febrile neutropenia considered related to treatment). Drug-related SAEs are summarized in Table 6. The most common drug-related SAE was febrile neutropenia (*n* = 6; 9.4%).

**Table 5:** Serious adverse events in patients treated with trastuzumab deruxtecan

	Total (*N* = 64)
SAEs	17 (26.6)
Drug-related SAEs	10 (15.6)
SAEs^a^ leading to drug withdrawal^b^	4 (6.3)
Drug-related SAEs leading to drug withdrawal^b^	3 (4.7)
SAEs^a^ leading to dose reduction^b^	5 (7.8)
Drug-related SAEs leading to dose reduction^b^	5 (7.8)
SAEs^a^ leading to drug interruption^b^	5 (7.8)
Drug-related SAEs leading to drug interruption^b^	4 (6.3)
SAEs^a^ leading to death	6 (9.4)
Drug-related SAEs leading to death	1 (1.6)

Data are shown as *n* (%)

^a^The adverse events collected were limited to potential interstitial lung disease and pneumonitis events (any severity or grade), liver-related events potentially meeting Hy’s Law criteria (any severity or grade), SAEs, and overdose

^b^Drug withdrawals, drug reductions, drug interruptions were recorded as the most severe action taken (i.e., following the priority of “drug withdrawal > drug interruption > drug reductions”)

SAEs, serious adverse events

**Table 6:** Serious drug-related adverse events in patients treated with trastuzumab deruxtecan (MedDRA Preferred Term)

	Total (*N* = 64)
Drug-related SAEs	10 (15.6)
Febrile neutropenia	6 (9.4)
Diarrhea	2 (3.1)
Pneumonitis	2 (3.1)
Anemia	1 (1.6)
Colitis	1 (1.6)
Enteritis	1 (1.6)
Hepatic encephalopathy	1 (1.6)
Nausea	1 (1.6)
Platelet count decreased	1 (1.6)
Sepsis	1 (1.6)
Upper gastrointestinal hemorrhage	1 (1.6)

Data are shown as *n* (%)

*MedDRA* medical dictionary for regulatory activities version 23.1; *SAEs* serious adverse events

The occurrence of drug-related ILD is tabulated in Table 7. Among the four patients with suspected ILD, three patients (Grade 1, Grade 2, and Grade 3: all *n* = 1) had drug-related ILD as determined by the external Adjudication Committee. No Grade 4 or higher events were reported. Potential Hy’s Law liver-related events occurred in three patients. However, these events were judged not to have met Hy’s Law criteria as there was an alternate cause present in all cases (bile stent obstruction, inflammation from colitis spread to the liver, and liver metastasis due to disease progression); thus, the reporting physician concluded that the events were not drug related.

**Table 7:** Determination of interstitial lung disease reported by the Adjudication Committee in patients treated with trastuzumab deruxtecan

ILD outcome	CTCAE grade reported by Adjudication Committee			
	Grade 1	Grade 2	Grade 3	Total
Investigator suspected ILD	–	–	–	4 (6.3)
Independently adjudicated as not ILD	–	–	–	1 (1.6)
Independently adjudicated as drug-related ILD	1 (1.6)	1 (1.6)	1 (1.6)	3 (4.7)

Data are shown as *n* (%)

No ILD events of Grade 4 or Grade 5 were reported

The Adjudication Committee assigned grades only to events that were determined to be ILD

*CTCAE* common terminology criteria for adverse events; *ILD* interstitial lung disease

## Discussion

This expanded-access study evaluated the safety of T-DXd in 64 patients with locally advanced or metastatic HER2-positive gastric or GEJ adenocarcinoma. Our study provided the opportunity for these patients to access T-DXd treatment prior to marketing approval in Japan.

The EAP system in Japan was established by the Japanese MHLW in 2016 [21, 22]; thus, there are relatively fewer examples of its use in Japan than in Europe and the USA [20]. The Japanese EAP system requires careful consideration of the disease stage, severity, and the drug’s potential for complications to ensure the safety of treated patients. Furthermore, expanded-access studies can only be conducted at institutions participating in the main trial and with sufficient prior experience with the study drug and any potential adverse reactions [22]. These studies are intended to both maximize the potential benefits for patients and bridge the gap between clinical trials and post-approval availability. In this expanded-access study, sites that overlapped with those in the DG-01 trial were selected to shorten the time to study initiation as much as possible, while also streamlining the design and taking regional characteristics into consideration. With the cooperation of the medical institutions and other relevant vendors, the expanded-access study was initiated rapidly, providing patients with the opportunity to receive T-DXd for at least 6 months before the drug was approved.

The Japanese MHLW requires that expanded-access studies follow a similar protocol to the corresponding clinical trial, while not interfering with the conduct of the clinical trial, and that the expanded-access study prioritize the safety of the patients [22]. Thus, it was equally important to give due consideration to ensuring patient safety, and also to ensure that the development of T-DXd in the related phase II DG-01 clinical trial was not adversely affected. Considering the limited evidence for the efficacy and safety of T-DXd for the treatment of previously treated HER2-positive advanced gastric cancer patients when this expanded-access study was initiated, this expanded-access study was conducted with most of the same eligibility criteria set in the phase II DG-01 trial regarding bone marrow, renal, hepatic, cardiac function, and performance status [18]. However, some of the eligibility criteria were modified in this expanded-access study: for example, patients without measurable lesions, and patients with confirmed progression on both irinotecan and paclitaxel were included. Furthermore, HER2 status in this expanded-access study could be determined from diagnostic results obtained from local laboratories at each hospital, whereas the DG-01 trial stipulated that HER2 status must be assessed by a central laboratory. The patient characteristics between the DG-01 trial and this expanded-access study were similar in terms of age and sex; however, the population in the expanded-access study included more patients who had received a greater number of previous cancer treatments than the DG-01 trial population [18]. In addition, our expanded-access study allowed patients who were assigned to the physician’s choice arm in the DG-01 trial; two patients from the physician’s choice cohort received T-DXd in this expanded-access study. These patients had the option to continue treatment at the end of the expanded-access study and following marketing approval. Thus, our expanded-access study provided T-DXd to patients who did not receive T-DXd in the phase II DG-01 trial and enabled patients to benefit from the continued T-DXd treatment. Most patients who discontinued treatment received the commercial drug of their own choice after this expanded-access study ended, thereby bridging the gap between clinical trials and post-approval availability.

The AEs reported in this expanded-access study were generally manageable and tolerable. As the dose was reduced in 27/64 patients (42.2%), physicians should modify the dose of T-DXd according to the patient’s condition and tolerability to mitigate AEs. It must be noted that the DG-01 trial and this expanded-access study were different in terms of treatment duration and data collection, which precludes direct comparisons. The median duration of T-DXd treatment in this expanded-access study was 2.7 months (range, 0.7–6.2), which is shorter compared with the DG-01 trial [18]; this may partly explain the discrepancy in the observed SAE frequencies between this study and the DG-01 trial (26.6% and 44.0%, respectively). In addition, the observed frequencies of drug-related SAEs in this expanded-access study and the DG-01 were 15.6% and 21.6%, respectively [18]. The reason for the difference in treatment duration is that this expanded-access study was conducted for only a short period until marketing approval in Japan. Furthermore, the data included many patients who had received fewer T-DXd administrations, including patients receiving only one administration just before the end of this expanded-access study. A comparison of SAEs with T-DXd treatment is challenging as SAE frequencies vary between trials, even among trials of patients with the same type of cancer [18, 23–28]. Thus, it is difficult to reach any conclusions regarding differences in SAE frequency between this expanded-access study and other T-DXd clinical trials because of differences in T-DXd dose, treatment line, treatment duration, and cancer type [18, 23–28]. However, the results of this expanded-access study are generally consistent with the safety results as identifying recommended dose in HER2-positive gastric cancer [29]. In addition, the observed safety profile of T-DXd in this expanded-access study was similar to the profile observed in previous clinical trials regarding AE type and severity; no new safety concerns were identified despite the relatively short study duration.

We acknowledge the limitations of the expanded-access study. In accordance with the Japanese guidelines for EAPs [21], the primary focus of this expanded-access study was to provide patients with access to T-DXd, and we did not comprehensively evaluate the safety or efficacy of T-DXd. Therefore, there were no biomarker assessments, including microsatellite instability assessments, circulating tumor DNA, and HER2 amplification detection, and not all AEs and clinical outcomes were reported on the CRFs. AEs outside of the scope of interest for the expanded-access study were not included on the CRFs; therefore, not all AEs were reported. Only SAEs, all potential cases of ILD/pneumonitis, all liver-related events, and all cases of overdose were reported. In addition, during the study period, the number of patients with progressive disease as assessed by a physician was 19/64 (29.7%), but the limited duration prior to study termination and the limited data provided in the CRFs (such as presence or absence of measured lesions) precluded any further inferences regarding clinical effectiveness.

In conclusion, this expanded-access study provided T-DXd to patients with locally advanced or metastatic HER2-positive gastric and GEJ adenocarcinoma prior to marketing approval of T-DXd in Japan. No unexpected SAEs or other significant events were reported, and no new safety concerns were identified.

## References

[1] Park DI, Yun JW, Park JH (2006). HER-2/neu amplification is an independent prognostic factor in gastric cancer. Dig Dis Sci.

[2] Kim KC, Koh YW, Chang HM (2011). Evaluation of HER2 protein expression in gastric carcinomas: comparative analysis of 1,414 cases of whole-tissue sections and 595 cases of tissue microarrays. Ann Surg Oncol.

[3] Cho EY, Park K, Do I (2013). Heterogeneity of ERBB2 in gastric carcinomas: a study of tissue microarray and matched primary and metastatic carcinomas. Mod Pathol.

[4] Shan L, Ying J, Lu N (2013). HER2 expression and relevant clinicopathological features in gastric and gastroesophageal junction adenocarcinoma in a Chinese population. Diagn Pathol.

[5] Yano T, Doi T, Ohtsu A (2006). Comparison of HER2 gene amplification assessed by fluorescence in situ hybridization and HER2 protein expression assessed by immunohistochemistry in gastric cancer. Oncol Rep.

[6] Yoon HH, Shi Q, Sukov WR (2012). Association of HER2/ErbB2 expression and gene amplification with pathologic features and prognosis in esophageal adenocarcinomas. Clin Cancer Res.

[7] Yan M, Parker BA, Schwab R (2014). HER2 aberrations in cancer: implications for therapy. Cancer Treat Rev.

[8] Moasser MM (2007). The oncogene HER2: its signaling and transforming functions and its role in human cancer pathogenesis. Oncogene.

[9] Gerson JN, Skariah S, Denlinger CS (2017). Perspectives of HER2-targeting in gastric and esophageal cancer. Expert Opin Investig Drugs.

[10] National Comprehensive Cancer Network (2022) NCCN Clinical Practice Guidelines in Oncology: Gastric Cancer version 2.2022. Available at: https://www.nccn.org/professionals/physician_gls/pdf/gastric.pdf. Accessed August 2023. 10.6004/jnccn.2022.0008 35130500

[11] Lordick F, Carneiro F, Cascinu S (2022). Gastric cancer: ESMO Clinical Practice Guideline for diagnosis, treatment and follow-up. Ann Oncol.

[12] Association JGC (2021). Gastric cancer treatment guidelines for physicians (in Japanese).

[13] Ogitani Y, Aida T, Hagihara K (2016). DS-8201a, A novel HER2-targeting ADC with a novel DNA topoisomerase I inhibitor, demonstrates a promising antitumor efficacy with differentiation from T-DM1. Clin Cancer Res.

[14] Ogitani Y, Hagihara K, Oitate M (2016). Bystander killing effect of DS-8201a, a novel anti-human epidermal growth factor receptor 2 antibody-drug conjugate, in tumors with human epidermal growth factor receptor 2 heterogeneity. Cancer Sci.

[15] Daiichi Sankyo Co., Ltd. (2022) Trastuzumab deruxtecan (Enhertu) for intravenous drip infusion [Japanese package insert]. (in Japanese). Available at: https://www.data-index.co.jp/dragdata/pdf/4/430574_4291452D1029_1_07.pdf. Accessed August 2023.

[16] Daiichi Sankyo, Inc. (2022) Trastuzumab deruxtecan (Enhertu [fam-trastuzumab deruxtecan-nxki]) for injection, for intravenous use [US prescribing information]. Available at: https://daiichisankyo.us/prescribing-information-portlet/getPIContent?productName=Enhertu&inline=true. Accessed August 2023.

[17] Daiichi Sankyo Europe GmbH. (2021) Trastuzumab deruxtecan (Enhertu) powder for concentrate for solution for infusion [EU summary of product characteristics]. Available at: https://www.ema.europa.eu/en/documents/product-information/enhertu-epar-product-information_en.pdf. Accessed August 2023.

[18] Shitara K, Bang YJ, Iwasa S (2020). Trastuzumab deruxtecan in previously treated HER2-positive gastric cancer. N Engl J Med.

[19] Doi T, Shitara K, Naito Y (2017). Safety, pharmacokinetics, and antitumour activity of trastuzumab deruxtecan (DS-8201), a HER2-targeting antibody-drug conjugate, in patients with advanced breast and gastric or gastro-oesophageal tumours: a phase 1 dose-escalation study. Lancet Oncol.

[20] Maeda H, Uchida M, Kusano M (2022). Characteristics of the compassionate use program in Japan: an analysis of expanded access clinical trials from 2016 to 2021. Clin Pharmacol Ther.

[21] Japan Pharmaceutical Manufacturers Association. Pharmaceutical Administration and Regulations in Japan 2020. Available at: https://www.jpma.or.jp/english/about/parj/eki4g600000078c0-att/2020.pdf. Accessed August 2023.

[22] Japan Ministry of Health and Welfare. (2016) Clinical trials conducted on ethical grounds. Available at: https://www.pmda.go.jp/files/000227843.pdf. Accessed August 2023.

[23] Modi S, Saura C, Yamashita T (2019). Trastuzumab deruxtecan in previously treated HER2-positive breast cancer. N Engl J Med.

[24] André F, Hee Park Y, Kim SB (2023). Trastuzumab deruxtecan versus treatment of physician's choice in patients with HER2-positive metastatic breast cancer (DESTINY-Breast02): a randomised, open-label, multicentre, phase 3 trial. Lancet.

[25] Cortés J, Kim SB, Chung WP (2022). Trastuzumab deruxtecan versus trastuzumab emtansine for breast cancer. N Engl J Med.

[26] Modi S, Jacot W, Yamashita T (2022). Trastuzumab deruxtecan in previously treated HER2-low advanced breast cancer. N Engl J Med.

[27] Li BT, Smit EF, Goto Y (2022). Trastuzumab deruxtecan in HER2-mutant non-small-cell lung cancer. N Engl J Med.

[28] Yoshino T, Di Bartolomeo M, Raghav K (2023). Final results of DESTINY-CRC01 investigating trastuzumab deruxtecan in patients with HER2-expressing metastatic colorectal cancer. Nat Commun.

[29] Yoshihara K, Kobayashi Y, Endo S (2023). Trastuzumab deruxtecan dosing in human epidermal growth factor receptor 2-positive gastric cancer: population pharmacokinetic modeling and exposure–response analysis. J Clin Pharmacol (in press).

